# The use of red flags during the referral chain of patients surgically treated for symptomatic spinal metastases

**DOI:** 10.1093/nop/npad013

**Published:** 2023-03-07

**Authors:** Floris R van Tol, Isabelle M L P Kamm, Anne L Versteeg, Karijn P M Suijkerbuijk, Helena M Verkooijen, Cumher Oner, Jorrit-Jan Verlaan

**Affiliations:** Department of Orthopaedic Surgery, UMC Utrecht, Heidelberglaan 100, 3584 CX Utrecht, the Netherlands; Department of Orthopaedic Surgery, UMC Utrecht, Heidelberglaan 100, 3584 CX Utrecht, the Netherlands; Imaging Division, UMC Utrecht, Heidelberglaan 100, 3584 CX Utrecht, the Netherlands; University of Toronto, 27 King’s College Cir, Toronto, ON M5S, Canada; Department of Medical Oncology, UMC Utrecht, Heidelberglaan 100, 3584 CX Utrecht, the Netherlands; Imaging Division, UMC Utrecht, Heidelberglaan 100, 3584 CX Utrecht, the Netherlands; Department of Orthopaedic Surgery, UMC Utrecht, Heidelberglaan 100, 3584 CX Utrecht, the Netherlands; Department of Orthopaedic Surgery, UMC Utrecht, Heidelberglaan 100, 3584 CX Utrecht, the Netherlands

**Keywords:** delay, metastatic spinal disease, red flags, spinal metastases, surgery

## Abstract

**Background:**

The use of so-called “red flags” may be beneficial in identifying patients with metastatic spinal disease. This study examined the utility and efficacy of these red flags in the referral chain of patients surgically treated for spinal metastases.

**Methods:**

The referral chains from the onset of symptoms until surgical treatment for all patients receiving surgery for spinal metastases between March 2009 and December 2020 were reconstructed. The documentation of red flags, as defined by the Dutch National Guideline on Metastatic Spinal Disease, was assessed for each healthcare provider involved.

**Results:**

A total of 389 patients were included in the study. On average, 33.3% of red flags were documented as present, 3.6% were documented as absent, and 63.1% were undocumented. A higher rate of red flags documented as present was associated with a longer time to diagnosis, but a shorter time to definitive treatment by a spine surgeon. Moreover, red flags were documented as present more often in patients who developed neurological symptoms at any point during the referral chain than those who remained neurologically intact.

**Conclusions:**

The association of red flags with developing neurological deficits highlights their significance in clinical assessment. However, the presence of red flags was not found to decrease delays prior to referral to a spine surgeon, indicating that their relevance is currently not sufficiently recognized by healthcare providers. Raising awareness of symptoms indicative of spinal metastases may expedite timely (surgical) treatment and thus improve treatment outcome.

As metastatic spinal disease progresses, patients may experience mechanical pain, radiculopathy, or neurological deficits due to epidural spinal cord compression.^1^ Early diagnosis and treatment are critical in avoiding irreversible neurological damage and achieving the best clinical outcome.^2–4^

Patients presenting with back pain as a symptom of advanced cancer often experience a delay of several weeks before being diagnosed with spinal metastases.^5^ This delay in diagnosis and treatment may be caused by patients not seeking medical attention in a timely manner and/or healthcare providers not recognizing the symptoms.^6^ Additionally, the high prevalence of nonspecific back pain in the general population makes it difficult for healthcare providers to distinguish between a serious underlying pathology (ie, advanced cancer) and/or a common benign condition.

To reduce delay in the diagnosis and treatment of spinal metastases, it is essential that both patients at risk and healthcare providers are cognizant of potential early warning signs. To this end, the Netherlands Comprehensive Cancer Organisation (IKNL) has identified 5 “red flags” that are indicative of metastatic spinal disease and included them in the Dutch national guideline on metastatic spinal disease.^7^ These red flags include: new onset of back pain, progressive back pain, nocturnal back pain, pain on palpation, and poor general health (eg, weight loss).^8^ Previous research has demonstrated that the presence of these red flags may increase the likelihood of metastatic spinal disease, with multiple red flags showing the highest predictive value.^9,10^ However, no studies have examined the use of red flags in clinical decision-making.

The primary aim of this study was to evaluate the documentation of red flags by Dutch healthcare providers for patients with and without a pre-existing malignancy across primary, secondary, and tertiary healthcare. As a secondary aim, we sought to better comprehend how the presence of red flags influences the referral chain by analyzing the association between red flags and delays in the referral chain. We hypothesized that the presence of red flags leads to expedited diagnosis, referral, and treatment. Additionally, we aimed to assess the clinical significance of red flags by correlating their presence to the development of neurological symptoms. We hypothesized that the presence of red flags is associated with faster neurological decline.

## Materials and Methods

### Patient Population and Study Design

The institutional review board approved a waiver for informed consent for this single-center retrospective cohort study (protocol no. 17-695/C). This study included patients aged 18 years and older who underwent primary surgery for symptomatic spinal metastases between March 2009 and December 2020 at the University Medical Centre Utrecht in the Netherlands. Additionally, patients with spinal localizations of hematological tumors, including multiple myeloma or malignant lymphoma, were included due to similarities in clinical presentation and surgical treatment compared with spinal metastases originating from solid tumors. Patients with sacral metastases or primary tumors of the spine and patients who received revision surgery were excluded from the study.

### Data-collection

Data on patient age, sex, tumor histology, history of cancer, neurological status, healthcare providers involved in the referral chain, levels of care (ie, primary, secondary, or tertiary care hospital), and documentation of the 5 (IKNL) red flags were extracted from patients’ electronic medical records. Neurological deficits were defined as grades A–D on the American Spinal Injury Association scale. Referral chains from the first symptoms related to metastatic spinal disease until surgery were reconstructed using data from the hospital’s medical records as well as the patients’ records kept by their general practitioners.

The interval between the onset of symptoms caused by the spinal metastasis/metastases and the start of definitive treatment was defined as the total delay, which could be divided into four sub-intervals: patient delay (period between the onset of symptoms and patient’s first contact with a healthcare provider), diagnostic delay (time interval between the first medical consultation and confirmed diagnosis of spinal metastases), referral delay (time interval between the diagnosis date and referral to the spine surgeon), and treatment delay (time interval between referral to the spine surgeon and surgical treatment). The combined diagnostic, referral, and treatment intervals were referred to as doctor delay.

### Outcomes

The medical records of each healthcare provider involved in the referral chain were examined to report the presence or absence of all 5 red flags (new onset of back pain, progressive back pain, nocturnal back pain, pain on palpation, and poor general health). The average number of red flags documented as present or absent, as well as the number of undocumented red flags were calculated for each patient across all healthcare providers.

### Statistical Analysis

Continuous variables were reported as means with standard deviations or medians and interquartile ranges, depending on their distribution. Categorical variables were reported as frequencies and percentages.

To compare the documentation of red flags between patients with and without previous malignancy and to compare the presence of red flags between patients who had developed neurological symptoms upon final consultation versus patients who had not, Mann–Whitney *U* tests were used (for non-normally distributed data). Sub-analyses using Mann–Whitney *U* tests were conducted to compare the percentage of documented red flags of each of the individual red flags between patients with pre-existing malignancy and patients without. To assess the relationship between the presence of red flags and different delay intervals, the percentage of red flags documented as present was stratified into five 20% intervals (for interpretability) and delay intervals between these strata were compared using Kruskal–Wallis analyses. Analyses were held to a significant threshold of *p* ≤ .05. All analyses were conducted using IBM SPSS Statistics, version 24 (IBM Corp).

## Results

**Figure 1.**
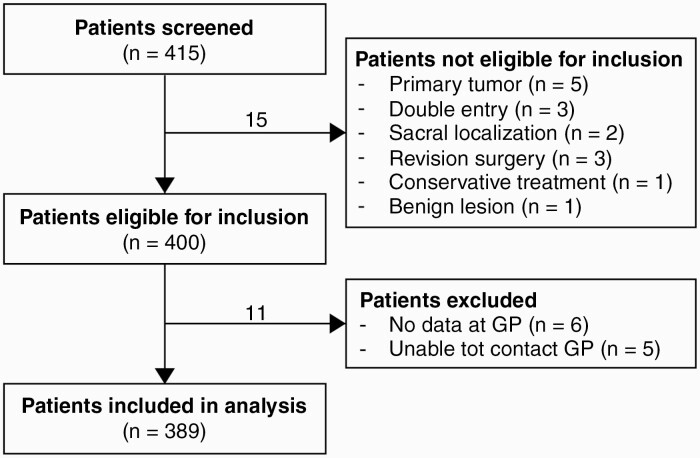
Flowchart of patient inclusion and exclusion.

A total of 400 patients who underwent surgery for symptomatic spinal metastases within the study period met the inclusion criteria. In 11 patients, no data could be retrieved from their general practitioner (*n* = 11), leaving a total of 389 patients to be included in the current study (Figure 1).

Table 1 provides the characteristics of all 389 patients, which comprised 231 patients with a pre-existing malignancy and 158 patients without. Overall, 218 (56.0%) of patients were male. The mean age at time of surgery was 62.3 years (SD ± 11.5 years). The most common primary tumors were urogenital (21.9%), hematological (19.8%), and breast cancer (18.8%). A median of 2 (IQR: 2–3) healthcare providers were involved in the referral chain before referral to the spine surgeon. Of all patients, 147 (37.8%) had developed neurological symptoms before definitive treatment, while the remaining 242 (62.2%) were neurologically intact at the final consultation.

**Table 1.** Baseline characteristics

Characteristic	All patients (*N* = 389)
**Age at surgery (years), *mean* *(SD)***	62.3 (11.5)
**Sex, male, *n (%)***	218 (56.0%)
**Tumor histology, *n (%)***	
Urogenital	85 (21.9%)
Hematological	77 (19.8%)
Breast	73 (18.8%)
Lung	58 (14.9%)
Gastrointestinal	25 (6.4%)
Gynecologic	6 (1.5%)
Other	56 (14.4%)
Unknown	9 (2.3%)
**Number of HCPs involved before treatment, *n (%)***	
One	78 (20.1%)
Two	149 (38.8%)
Three	115 (29.6%)
Four	34 (8.7%)
Five or more	13 (3.3%)
**Type of first HCP involved, *n (%)***	
General practitioner	253 (65.0%)
Emergency unit	30 (7.7%)
Oncological caregiver	81 (20.8%)
Radiation oncologist	6 (1.5%)
Orthopedic surgeon	4 (1.0%)
Neurologist	9 (2.3%)
Other	6 (1.5%)
**Level of care on entry, *n (%)***	
Primary	253 (65.0%)
Secondary	91 (23.4%)
Tertiary	45 (11.6%)

SD: standard deviation, HCP: healthcare provider.

### Documentation of Red Flags

On average, 36.9% of the potential 5 red flags per healthcare provider were documented prior to the diagnosis of spinal metastases, with 90.3% documented as present. A statistically significant difference (*p* = .028) was found in the documentation of red flags between patients without a history of cancer (median 40%; IQR 32%–45%) and those with a pre-existing malignant diagnosis (median 35%; IQR 28%–40%). Additionally, documentation of red flags was lowest in primary care (32.2%) compared with secondary (35.6%), and tertiary care (40.4%), these data are summarized in Table 2. Of the red flags, new onset of back pain was the most frequently documented (in 97.8% of healthcare providers at any time point), whereas nocturnal back pain and poor general health were documented the least (8.5% and 9.2%, respectively), as summarized in Table 3.

**Table 2.** Mean percentage of documented red flags stratified for patients with and without a pre-existing malignancy and the type of care

Type of care	Total (*N* = 389)	Pre-existing malignancy (*n* = 231)	No pre-existing malignancy (*n* = 158)
**% red flags documented**			
Primary care	32.2%	30.2%	33.8%
Secondary care	35.6%	32.8%	38.8%
Tertiary care	40.4%	39.8%	41.2%

**Table 3.** Mean percentage of individual documented red flags (as either present or absent)

Red flag	Documented
Nocturnal back pain	8.5%
New back pain	97.8%
Progressive back pain	50.2%
Pain on palpation	18.7%
Poor general health	9.2%

### Guidance of the Referral Process through Red Flags

The presence of red flags was significantly correlated with longer diagnostic delay and shorter treatment delay, as demonstrated by Table 4. No significant association between the presence of red flags and any other delay intervals was identified.

**Table 4.** Kruskal–Wallis test comparing the median (IQR) delay (in days) across five different patient categories, based on the percentage of red flags documented as present out of all documented red flags

Delay interval	% of red flags documented as present			
	0%–60% (*n* = 15)	61%–80% (*n* = 47)	81%–100% (*n* = 314)	*p*-value
Total delay	59	130	97	.078
Patient delay	14	15	19.5	.652
Doctor delay	37	71	53	.120
Diagnostic delay	3	47	22	<.001^*^
Referral delay	4	11	7	.290
Treatment delay	16	8	6	.013^*^

^*^ *p* ≤ .05.

### Red Flags as Predictors for Neurological Deficits

In 260 patients (66.8%), all documented red flags were present. Of those who had developed neurological deficits at any point in the referral chain, all documented red flags were present in 109 patients (74.7%). Conversely, all documented red flags were present in 151 (62.7%) patients who remained neurologically intact. The presence of red flags at any point during the referral chain was significantly more prevalent among those who developed neurological deficits before definitive treatment compared with patients who remained neurologically intact (*p* = .006).

## Discussion

This study aimed to investigate the use and utility of a set of 5 red flags in patients with symptomatic spinal metastases who underwent surgical intervention. Despite the opportunity for all healthcare providers to document 5 red flags (regardless whether any of the red flags were actually raised), only 36.9% were recorded. This suggests that clinicians may not be sufficiently aware of the symptoms indicating severe spinal pathology and as a result may not ask for (the presence of) red flags. Contrary to the initial hypothesis, a higher number of red flags documented as present did not lead to a faster diagnosis and referral. However, a higher percentage of red flags documented as present was associated with an increased risk of developing neurological deficits before definitive treatment, confirming the second research hypothesis. These findings emphasize the need for systematic use of red flags, as patient outcome is largely dependent upon neurological status before treatment.^11^

Back pain is a frequent complaint among primary care patients, with up to 70% of people experiencing an episode of (nonspecific) back pain in their lifetime.^12^ While it is generally self-limiting, a small percentage of patients may have a serious underlying condition such as metastatic spinal disease. Studies have shown that the prevalence of spinal malignancy in patients with back pain ranges from 0% to 0.7%, while it increases to 7.0% in hospital settings.^13,14^ The current study found that 36.9% of red flags were documented, a rate similar to that reported in a study by Ferguson et al. (33%),^15^ but lower than that reported by Leerar et al. (63.7%).^16^ Both studies, however, did not focus solely on red flags indicative of spinal metastases, but also examined red flags indicative of fractures or infectious disease. Additionally, the studies by Leerar et al. and Ferguson et al. examined the documentation of red flags by physical therapists, while the current study looked at the percentage of documented red flags by general practitioners and hospital-based medical specialists. Ferguson et al. found that the documentation of red flags improved from 33% to 65% after an active intervention, consisting of the development and implementation of action plans, was conducted. The results from their study clearly underline the potential for education to help raise awareness and have doctors explicitly ask for the presence of red flags. It has previously been suggested that despite the inclusion of red flags in clinical guidelines, patients are not always evaluated in line with the recommendations from the guidelines.^17^ This is also supported by a study of Amorin-Woods et al. who demonstrated that only 5% of medical practitioners followed the recommendations of clinical guidelines to identify red flags in patients with low back pain.^18^

Healthcare providers documented fewer red flags in patients with a pre-existing malignancy compared with those without. Patients with a pre-existing malignancy may be more likely to visit their oncology professional, who may be less inclined to screen for red flags associated with spinal metastases as the combination of a pre-existing malignancy and back pain may already warrant further diagnostics. Additionally, these patients may have more other complaints or side effects of cancer treatments, which could reduce the focus on back pain and associated symptoms. Moreover, symptoms such as poor general health are not specific to spinal metastases and may not raise immediate new concerns or suspicions in oncological patients. In contrast, patients without a pre-existing malignancy will likely visit their general practitioner where an extensive medical history and physical examination focusing on the primary complaint, that is, back pain, takes place.

The current investigation of the documentation of red flags revealed that healthcare providers documented specific red flags such as new onset of back pain (97.8%) more often than symptoms like nocturnal pain and poor general health (8.5% and 9.2%, respectively) regardless of whether they were documented as present or absent. This discrepancy in documentation could be due to the fact that new onset of back pain is frequently the primary reason for seeking medical aid, whereas poor general health is usually reported in later stages of malignant disease.^19,20^ These results suggest that healthcare providers may not be screening for poor general health or nocturnal pain during the initial consultation, which could explain the low documentation rate observed in the current study.

In the current study, it was found that treatment delay (ie, the interval between referral to the spine surgeon and surgical treatment) was significantly reduced as the number of red flags documented as present increased. Interestingly, an increased presence of red flags was associated with a longer diagnostic delay (ie, the interval between the first medical consultation and confirmed diagnosis of spinal metastases), indicating a potential issue in how red flags are currently being used to aid healthcare providers in their clinical decision-making. In the presence of red flags, patients would preferably be referred and/or diagnosed in an accelerated manner. It was also found that presence of red flags was not significantly associated with a reduction in patient delay. A possible explanation for this finding may include patients who have a known pre-existing malignancy not properly being informed about the existence and importance of red flags or not reporting on them. Furthermore, while for oncological patients, education about red flags as warning signs for spinal metastases could potentially reduce patient delay, this is not feasible for those unaware of an underlying malignancy. Another potential explanation is that red flags are simply less frequently present in the early stages of the referral chain.

Since this study only included patients with confirmed metastatic spinal disease, no conclusions can be drawn on the specificity or sensitivity of the described red flags. However, in the current study, the presence of red flags was associated with neurological deterioration prior to definitive treatment. This finding suggests that the presence of (multiple) red flags may be indicative of a more advanced stage of metastatic spinal disease, emphasizing the importance of red flags for a timely diagnosis, referral, and treatment.

This study has limitations that need to be taken into account when interpreting the results. First, the current study was retrospective with its inherent risks of bias. Only red flags that were documented in the electronic patient records could be considered, potentially leading to an underestimation of red flags that were present. Healthcare providers may not have documented the red flags that were absent in their patient, causing a potential discrepancy between red flags that were documented and the red flags that were discussed but not recorded during medical consultation. Nevertheless, it is likely that the underreporting of red flags was consistent across all patients included and thus would not have influenced the conclusions. Moreover, a prospective study on red flags for metastatic spinal disease would be difficult to conduct due to the low incidence of metastatic spinal disease in the general population, which adds to the value of the current study. Second, only patients who received surgical treatment were included in this study; those who underwent radiotherapy or systemic treatment were not included. Healthcare providers may have been more likely to screen for red flags in surgical patients, as they are generally more severely affected. This may have resulted in a higher proportion of red flags documented as present in these patients. Lastly, this was a single-center study, thus the results may not be applicable to other locations. Nonetheless, data were collected from referral chains throughout the entire region, suggesting that the results are representative for the Netherlands.

In conclusion, the current study provides insight into the use of red flags in the referral process of patients undergoing surgical treatment for symptomatic spinal metastases. Documentation of red flags among healthcare providers was limited, and the presence of red flags was not associated with shorter delays prior to referral to the definitive caregiver. However, a greater proportion of red flags documented as present was associated with poorer neurological outcomes. This finding supports the notion red flags are important in clinical decision-making and suggests that promoting the appropriate use of red flags may help reduce delays and clinical outcomes for patients with metastatic spinal disease.
